# Impact of salvage cytotoxic chemotherapy on prognosis in patients with recurrence after radical cystectomy: a multi-institutional retrospective study

**DOI:** 10.1186/s12894-022-01026-3

**Published:** 2022-05-13

**Authors:** Dai Koguchi, Kazumasa Matsumoto, Masaomi Ikeda, Yoshinori Taoka, Takahiro Hirayama, Yasukiyo Murakami, Takuji Utsunomiya, Daisuke Matsuda, Norihiko Okuno, Akira Irie, Masatsugu Iwamura

**Affiliations:** 1 Department of Urology, Kitasato University School of Medicine, 1-15-1 Kitasato Minami-ku Sagamihara, Kanagawa, 252-0374 Japan; 2 Department of Urology, Kitasato University Medical Center, Saitama, Japan; 3 Department of Urology, Kitasato University Kitasato Institute Hospital, Tokyo, Japan; 4 Department of Urology, Kanagawa Prefectural Federation of Agricultural Cooperatives for Health and Welfare Sagamihara Kyodo Hospital, Kanagawa, Japan; 5 Department of Urology, Higashiyamato Hospital, Tokyo, Japan; 6 Department of Urology, National Hospital Organization Sagamihara Hospital, Kanagawa, Japan

**Keywords:** Bladder cancer, Radical cystectomy, Salvage cytotoxic chemotherapy, Prognosis

## Abstract

**Background:**

In patients experiencing disease recurrence after radical cystectomy (RC) for bladder cancer, data about the impact of clinicopathologic factors, including salvage treatment using cytotoxic chemotherapy, on the survival are scarce. We investigated the prognostic value of clinicopathologic factors and the treatment effect of salvage cytotoxic chemotherapy (SC) in such patients.

**Methods:**

In this retrospective study, we evaluated the clinical data for 86 patients who experienced recurrence after RC. Administration of SC or of best supportive care (BSC) was determined in consultation with the urologist in charge and in accordance with each patient’s performance status, wishes for treatment, and renal function. Statistical analyses explored for prognostic factors and evaluated the treatment effect of SC compared with BSC in terms of cancer-specific survival (CSS).

**Results:**

Multivariate analyses showed that liver metastasis after RC (hazard ratio [HR] 2.13; 95% confidence interval [CI] 1.17 to 3.85; *P* = 0.01) and locally advanced disease at RC (HR 1.92; 95% CI 1.06 to 3.46; *P* = 0.03) are independent risk factors for worse CSS in patients experiencing recurrence after RC. In a risk stratification model, patients were assigned to one of two groups based on liver metastasis and locally advanced stage. In the high-risk group, which included 68 patients with 1–2 risk factors, CSS was significantly better for patients receiving SC than for those receiving BSC (median survival duration: 9.4 months vs. 2.4 months, *P* = 0.005). The therapeutic effect of SC was not related to a history of adjuvant chemotherapy.

**Conclusions:**

The present study indicated the potential value of 1st-line SC in patients experiencing recurrence after RC even with advanced features, such as liver metastasis after RC and locally advanced disease at RC.

## Background

Bladder cancer is the most common malignancy of the urinary tract and the 4th most common cancer in men [1]. Since the early 1990s, radical cystectomy (RC) has been the standard of care for patients with muscle-invasive and non-muscle-invasive bladder cancer that is refractory to intravesical therapy. In spite of progress in surgical techniques and an improved understanding of the role of pelvic lymphadenectomy, oncologic outcomes after RC are unfavorable; the cancer often recurs within the first 2–3 years, and only about one fifth of patients experiencing recurrence survive 5 years [2, 3]. Salvage treatment for recurrence after RC therefore remains a major challenge in daily clinical practice.

Historically, metastatic urothelial cancer (mUC) has been reported to be chemotherapy-sensitive. The combination methotrexate–vinblastine–doxorubicin–cisplatin (MVAC) in the late 1980s and the doublet gemcitabine–cisplatin (GC) in the late 1990s were associated with response rates in the range of 40–60% and a median overall survival of nearly 15 months in patients with mUC [4, 5]. Moreover, even in patients unfit to receive cisplatin, other regimens such as gemcitabine-containing chemotherapy have also been associated with acceptable results and response rates of 30%–40% [6, 7]. Cytotoxic chemotherapy (CC) has thus played a central role in the systemic treatment of mUC for more than 30 years.

Since about 2015, immune checkpoint inhibitors (ICIs) have revolutionized the treatment of mUC. In platinum-refractory advanced urothelial cancer, robust evidence has demonstrated improved overall survival after 2nd-line treatment with an ICI over CC alone [8, 9]. However, patients whose disease progresses after 1st-line CC experience a high symptom burden that causes rapid deterioration in physical function, often making them unfit for 2nd-line chemotherapy [10]. Then, very recently, the JAVELIN Bladder 100 phase III study showed significantly longer overall survival with 1st-line maintenance therapy using avelumab, a PD-L1 inhibitor, than with CC using a platinum-based regimen alone in patients with advanced urothelial cancer [8]. In particular, non-progression after prior CC was found to be an excellent clinical biomarker of better survival with maintenance therapy, reinforcing the value of 1st-line salvage cytotoxic chemotherapy (SC) for mUC. To date, data about the survival impact of clinicopathologic factors, including SC, in patients experiencing recurrence after RC have remained scarce, while factors prognostic for the development of disease recurrence after RC have been extensively studied [11, 12]. Hence, we investigated the effect of SC and the prognostic value of clinicopathologic factors in patients experiencing recurrence after RC.

## Methods

### Patient selection

We retrospectively reviewed clinical data for 361 patients who, between 1990 and 2015, underwent RC for bladder cancer at 6 hospitals affiliated with Kitasato University [13]. Patients with history of neoadjuvant chemotherapy (*n* = 75) were excluded, and 86 patients (30.1%, 86/286) had experienced recurrence after RC. Administration of either SC or best supportive care (BSC) was determined in consultation with the urologist in charge and in accordance with each patient’s performance status, wishes for treatment, renal function and prior history of adjuvant chemotherapy (AC) such as regimen of chemotherapy and treatment cycles. When cisplatin was administered to patients with impaired renal function, the dose was reduced by 25% for c*reatinine clearance of 46–60 ml/min, and* 50% for that of 31–45 ml/min. No patient in this cohort received any ICI. Our study was approved by the Institutional Review Boards at Kitasato University School of Medicine, Kitasato University Medical Center, Kitasato University Kitasato Institute Hospital, Kanagawa Prefectural Federation of Agricultural Cooperatives for Health and Welfare Sagamihara Kyodo Hospital, Higashiyamato Hospital and National Hospital Organization Sagamihara Hospital, including the request to waive documentation of informed consent (B15-25).

### Clinicopathologic evaluation

Data on patient characteristics collected from medical charts included age at recurrence of bladder cancer after RC; sex; post-RC pathology status (pT, pN, tumor grade, lymphovascular invasion, carcinoma in situ, and soft-tissue surgical margins); history of AC; history of SC; time to recurrence; recurrence sites; and mortality after recurrence. Tumors were graded using the 1973 World Health Organization grading system, and stage was assessed based on the 2002 TNM classification of malignant tumors [14, 15]. Surgical specimens were processed according to standard pathology procedures at each institution.

### Follow-up

Patients were generally followed every 3 months for the first 2 years after RC, then every 6 months for the next 3 years, and annually thereafter. Follow-up consisted of a physical examination, routine blood tests, and urinary cytology. Computed tomography and chest radiography were performed every 6 months for the first 2 years and annually thereafter. Bone scans were performed when clinical indications for disease progression were observed.

### Statistical analysis

Patients experiencing recurrence were divided into two groups: those who received SC and those who received BSC. Clinicopathologic factors were compared for the SC group and the BSC group. The chi-square test (or Fisher exact test, if appropriate) was used for categorical variables, and the Mann–Whitney *U *test, for continuous variables. Cancer-specific survival (CSS) after post-RC recurrence was estimated by the Kaplan–Meier method with the log-rank test. A multivariate analysis for CSS was performed using a Cox proportional hazards regression model, controlling for the effects of clinicopathologic factors. Based on independent risk factors for worse CSS revealed in the multivariate analyses, we constructed a risk-stratification model to evaluate the prognostic impact of SC on patients with those risk factors. All statistical analyses were performed in the Stata software application (version 13 for Windows: StataCorp LP, College Station, TX, USA). All *P* values are 2-sided, and *P* < 0.05 was considered statistically significant.

## Results

Table 1 shows the characteristics of the patients experiencing recurrence after RC. The study cohort consisted of 65 men (75.6%) and 21 women (24.4%) with a median age of 70 years at recurrence. Of those 86 patients, 38 (44.2%) received SC, and 48 (55.8%) received BSC. In terms of oncologic outcomes, 83.7% of the patients (*n* = 72) died from their cancer (SC: 89.5% [*n* = 34]; BSC: 79.2% [*n* = 38]), with a median time to recurrence of 12.0 months (interquartile range [IQR]: 4.7–24.5 months) and a median CSS of 4.7 months (IQR: 2.1–12.4 months). We observed no significant difference in clinicopathologic factors between the groups with the exception of the proportions of male and female patients.

**Table 1:** Characteristics of patients with either salvage cytotoxic chemotherapy (SC) or best supportive care (BSC)

Characteristic	BSC (*n* = 48)	SC (*n* = 38)	*P *value
Age, years [median [IQR])	71 (62–76)	69 (61–75)	0.71
Sex (*n* [%])			
Male	32 (66.6)	33 (86.8)	0.043
Female	16 (33.3)	5 (13.2)	
T Stage (*n* [%])			
≤ pT2	15 (31.2)	12 (31.6)	0.99
≥ pT3	31 (64.6)	25 (65.8)	
Unknown	2 (4.2)	1 (2.6)	
N Stage (*n* [%])			
pN0	31 (64.6)	25 (65.8)	0.91
≥ pN1	17 (35.4)	13 (34.2)	
Grade (*n* [%])			
G1/2	13 (27.1)	10 (26.3)	0.99
G3	31 (64.6)	24 (63.2)	
Unknown	4 (8.3)	4 (10.5)	
Lymphovascular invasion (*n* [%])			
Positive	29 (60.4)	25 (65.8)	0.43
Negative	17 (35.4)	10 (26.3)	
Unknown	2 (4.2)	3 (7.9)	
Carcinoma in situ (*n* [%])			
Positive	6 (12.5)	4 (10.5)	0.72
Negative	40 (83.3)	34 (89.5)	
Unknown	2 (4.2)	0	
Soft-tissue surgical margin (*n* [%])			
Positive	6 (12.5)	10 (28.9)	0.10
Negative	42 (87.5)	28 (71.1)	
Adjuvant chemotherapy (*n* [%])			
Yes	19 (39.6)	11 (72.7)	0.30
No	29 (60.4)	27 (27.3)	
Follow-up, months (median [IQR])	17.1 (9.0–39.3)	27.5 (14.1–40.8)	0.049

*IQR* interquartile range

The 152 recurrence sites observed were lymph node (*n* = 39, 25.6%), liver (*n* = 26, 17.1%), bone (*n* = 25, 16.4%), lung (*n* = 21, 13.8%), upper urinary tract (*n* = 17, 11.2%), peritoneum (*n* = 7, 4.6%), skin (*n* = 6, 3.9%), brain (*n* = 5, 3.3%), and others (*n* = 6, 3.9%). In the SC group, 82.8% of the 41 SC regimens administered were cisplatin-based: MVAC (*n* = 14, 34.1%), GC (*n* = 13, 31.7%), epirubicin–cisplatin (*n* = 6, 14.6%), and methotrexate–vincristine–cisplatin (*n* = 1, 2.4%). Others included gemcitabine–paclitaxel (*n* = 4, 9.8%), nedaplatin alone (*n* = 2, 4.9%), and gemcitabine alone (*n* = 1, 2.4%). Cisplatin was also dominant among the 30 AC regimens delivered: MVAC (*n* = 15, 50.0%), GC (*n* = 11, 36.7%), methotrexate–epirubicin–cisplatin (*n* = 2, 6.7%), gemcitabine–paclitaxel (*n* = 1, 3.3%), and carboplatin–etoposide (*n* = 1, 3.3%). Of the 38 patients who received SC, 35 (92.1%) received it in the 1st line, and 3 (7.9%), in the 2nd line. A median of 3 courses of both SC (IQR: 1–4 courses) and AC (IQR: 1–10 courses) were delivered during the follow-up period, and 86.7% of the patients given AC (*n* = 26 of 30) subsequently received the same regimen as SC (MVAC: 14/15; GC: 11/11; gemcitabine–paclitaxel: 1/1).

**Fig. 1:**
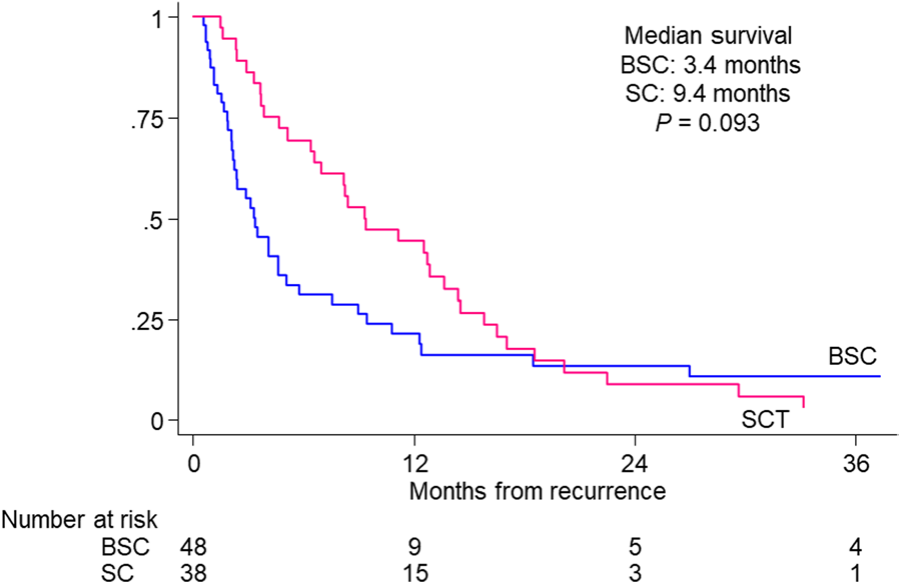
Kaplan–Meier analysis of CSS in patents receiving either SC or BSC

A Kaplan–Meier analysis showed that the median survival duration was 6 months longer for patients in the SC group than for those in BSC group, a nonsignificant difference (SC: 9.4 months; BSC: 3.4 months; Fig. 1). A multivariate analysis adjusted for the effects of clinicopathologic factors showed that liver metastasis (hazard ratio [HR] 2.13; 95% confidence interval [CI] 1.17 to 3.85; *P* = 0.01) and locally advanced disease (HR 1.92; 95% CI 1.06 to 3.46; *P* = 0.03) were independent risk factors for worse CSS (Table 2).

**Table 2:** Univariate and multivariate analyses for worse cancer-specific survival

Variable	Category	Univariate analysis			Multivariate analysis		
		HR	95% CI	*P* value	HR	95% CI	*P* value
Age	≥ 70	1.04	0.66–1.66	0.86	1.37	0.82–2.28	0.23
	≤ 69	1.0			1.0		
Sex	Female	1.36	0.80–2.30	0.25	1.51	0.82–2.79	0.19
	Male	1.0			1.0		
T stage	≥ pT3	1.47	0.88–2.46	0.14	1.92	1.06–3.46	0.03
	≤ pT2	1.0			1.0		
Metastasis							
Liver	Positive	1.68	1.02–2.77	0.041	2.13	1.17–3.85	0.01
	Negative	1.0			1.0		
Lung	Positive	1.21	0.72–2.05	0.47	1.57	0.85–2.91	0.15
	Negative	1.0			1.0		
Lymph node	Positive	0.89	0.56–1.42	0.63	0.90	0.53–1.52	0.69
	Negative	1.0			1.0		
Bone	Positive	0.89	0.53–1.48	0.66	0.90	0.48–1.69	0.75
	Negative	1.0			1.0		
AC	Positive	0.76	0.46–1.24	0.27	0.53	0.27–1.03	0.06
	Negative	1.0			1.0		
SC	Positive	0.67	0.42–1.07	0.096	0.72	0.40–1.29	0.27
	Negative	1.0			1.0		

*HR* hazard ratio, *CI* confidence interval, *TTR* time to recurrence, *RC* radical cystectomy, *AC* adjuvant chemotherapy, *SC* salvage cytotoxic chemotherapy

**Fig. 2:**
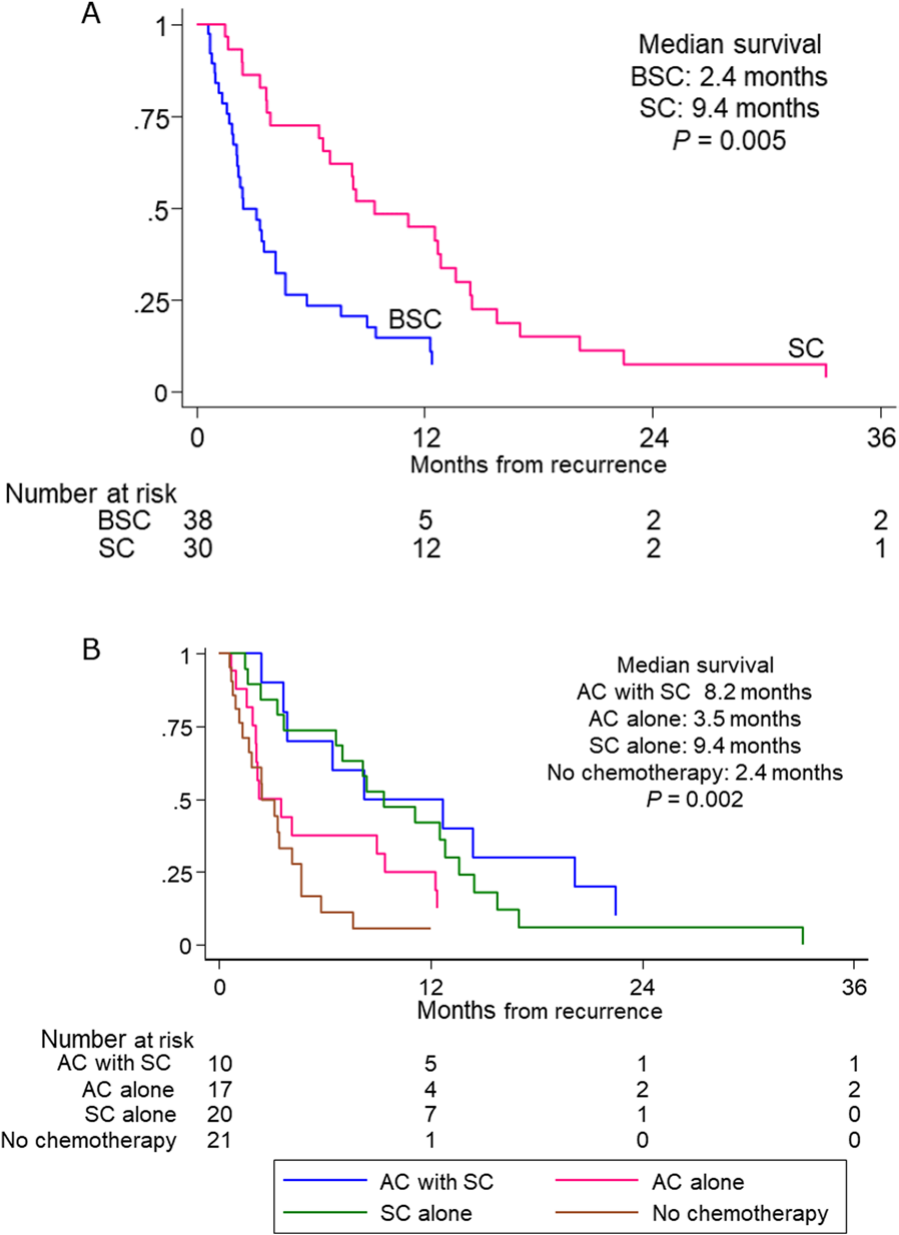
**A** Kaplan–Meier analysis of CSS in the high-risk group receiving either SC or BSC. **B** Kaplan–Meier analysis of CSS in the high-risk group based on history of AC and SC

In the risk stratification model, patients were assigned to one of two groups based on the presence of liver metastasis and locally advanced disease. The low-risk group, with neither risk factor, consisted of 18 patients, and the high-risk group, with either or both of the risk factors, consisted of 68 patients. In the low-risk group, a Kaplan–Meier analysis revealed no significant difference in CSS between patients receiving SC and those receiving BSC (*P* = 0.394). In the high-risk group, CSS was significantly better for patients receiving SC than for those receiving BSC, with median survival durations of 9.4 months and 2.4 months respectively (*P* = 0.005, Fig. 2A).

We also analyzed 4 treatment groups categorized according to history of SC and AC: patients receiving AC with SC (*n* = 11, 12.8%), those receiving AC alone (*n* = 19, 22.1%), those receiving SC alone (*n* = 27, 31.4%), and those receiving no chemotherapy (*n* = 29, 33.7%). Median interval from AC to SC was 9.5 months (IQR: 4.7–16.3 months). The baseline characteristics of the patients receiving (*n* = 30) and not receiving AC (*n* = 56) were not significantly different with respect to factors evaluated for the SC groups; however, a significantly greater proportion of patients receiving AC had locally advanced disease (86.2% [25/29] vs. 57.4% [31/54], *P* = 0.008) and lymphovascular invasion (81.5% [22/27] vs. 59.3% [32/54], *P* = 0.046). In the high-risk group, which was classified in the same way as for the analysis of patients receiving SC or BSC, the CSS was significantly different in the 4 groups. Regardless of AC history, survival duration was longer for patients who received SC than for those who did not receive SC (median CSS: 8.2 months [AC and SC], 9.4 months [SC alone], 3.5 months [AC alone], and 2.4 months [no chemotherapy]; *P* = 0.002; Fig. 2B). CSS was comparable in the two treatment groups receiving SC, being without a significant difference.

## Discussion

Intensive research has set out to find predictors of recurrence after RC [16], but only a limited number of studies have investigated clinicopathologic factors in patients that were prognostic after recurrence post-RC [11, 12, 17, 18]. The latter studies also demonstrated the lethal nature of recurrence after RC, showing a median overall survival of about 6 months. However, most of the studies reported local salvage treatments such as radiation therapy despite that one of the primary reasons for post-RC mortality has been suggested to be micrometastasis at the time of RC [17–19]. Our study demonstrated that liver metastasis after RC and locally advanced disease at RC were independent risk factors for worse CSS in patients with the experiencing recurrence after RC. Furthermore, our analysis of systemic treatment with SC found that CSS was significantly better for high-risk patients receiving SC than for those receiving BSC, and that, compared with AC, SC played a more important role in the treatment of recurrence after RC in the high-risk group.

The beneficial impact of SC demonstrated in the present study accords with earlier studies focusing on patients recurring after RC. Unlike the earlier studies, though, our study quantified the prognostic value of SC in the high-risk group, whose median CSS was 7 months longer than that of similar patients not receiving SC [11, 18]. Those favorable results after use of SC even in aggressive disease might encourage clinicians to consider the therapeutic merits of SC even given the risk for deterioration in quality of life because of the potential toxicity. From a new perspective, the JAVELIN Bladder 100 trial highlighted the significant value of CC in the treatment of mUC; CC followed by a maintenance therapy with avelumab was associated with the longest median overall survival of 21.4 months from among all reported ICI settings, including mono therapy and ICI combined with CC. Notably, non-progression with 1st-line CC was found to be an excellent prognostic biomarker [9]. As shown in the present study, a recurrent symptom burden after 1st-line chemotherapy leads to only a small proportion of patients receiving 2nd-line therapy [10, 20], and so 1st-line SC should be worth reinvestigating even in the era of ICIs. Additionally, two recent retrospective studies showed favorable results after reuse of CC for the progression of mUC with ICI or enfortumab vedotin. One of the mechanisms could be a synergistic effect of CC with the post ICI or enfortumab vedotin immunological context [21, 22]. Given the current standard treatment sequence of ICI-enfortumab for mUC, CC might be also notable for the 4th-line treatment.

Additionally, we shed light on two intriguing therapeutic aspects of SC in relation to AC in the high-risk group. First, regardless of a history of AC, CSS was significantly better for patients receiving SC than for those receiving AC alone and no chemotherapy. Second, for patients receiving SC, we observed no significant differences between those with and without a history of AC, and there was also no significant differences between patients receiving AC alone and those receiving no chemotherapy. In other words, in the high-risk group, once a bladder cancer recurred after RC, AC was not associated with a survival benefit, and the impact of SC on CSS was greater than that of AC despite the fact that most patients receiving AC had received the same regimen as SC. This observation is supported by the largest AC study from the European Organisation for Research and Treatment of Cancer trial 30994 [23]. In patients with locally advanced disease or node positivity (pN1–3) proven by RC, AC (compared with SC) provided no OS benefit, despite a highly significant improvement in 5-year progression-free survival (47.6% with AC vs. 31.8% with SC, *P* < 0.0001). As long as acquired chemotherapeutic cross resistance remains a major clinical concern in urothelial cancer, switching from CC to other treatments might be one of the choices [24, 25]. Recent clinical trials of ICIs have tended to include patients whose disease recurred within 12 months after AC; the prognosis for patients who received both AC and SC in the present study might have been better if CC had been replaced by an ICI as salvage treatment [8, 26]. Currently, clinical trials to explore optimal combination of ICIs, CC, and DNA damage repair protein inhibitor or fibroblast growth factor inhibitor in mUC are ongoing [27, 28].

In terms of prognostic factors for mUC when initially treated with CC, consensus has been reached that liver metastasis in particular carries the worse prognostic value in multivariate analyses adjusted by clinicopathologic variables [29, 30]. On the other hand, no previous study in patients whose disease recurred after RC has evaluated survival differences by metastatic site in multivariate analyses [11, 12, 17, 18]. A possible mechanism for the prognostic value of liver metastasis might relate to non-coding RNAs, which mainly control lipid metabolism in the liver [31]. In the last few years, aberrant expression of non-coding RNAs has been found to be associated with worse prognosis in some cancers [32, 33]. Furthermore, evidence has been increasing that non-coding RNAs expression is associated with cisplatin resistance, especially in ovarian cancer, in which debulking surgery has been widely accepted into clinical practice [34]. Although a recent review of in vitro experiments showed such an association between non-coding RNAs and cisplatin resistance, the role of metastasectomy in bladder cancer remains unclear because of the lack of a randomized setting [35, 36]. As a meta-analysis showed that the therapeutic effect not only of CC but also of ICIs for liver metastasis from urothelial cancer seemed to be transient [37], surgical consolidation for metastasis might be a key to achieving long-term survival in selected conditions. In fact, a recent clinical review outlined potential surgical indications for surgical consolidation in mUC, including a single liver metastasis [38]. Large, and possibly prospective, clinical studies are required to verify appropriate patient selection.

Our study had some limitations. First, the study’s retrospective design and lack of randomization could have introduced bias in the patient selection process. Second, RC was performed by multiple surgeons, and management of the postoperative chemotherapy (such as treatment intensity) was decided by the doctor in charge of each case—differences that might have influenced our results. Third, the small number of patients in the low-risk group did not permit a fair evaluation of the statistical impact of SC in the group. However, a therapeutic effect of SC was demonstrated in the relatively larger high-risk group, making further studies possibly worth conducting to validate the benefit of SC in both the low- and high-risk groups. Finally, we omitted some patient characteristics such as smoking status that potentially affect prognosis in bladder cancer. However, we believe that a focus on the pathology findings, when combined with the SC treatment status, could provide the explanation for the differences in prognosis.

## Conclusions

The present study, with its focus on SC, demonstrated that liver metastasis after RC and locally advanced disease at RC were independent risk factors for worse CSS in patients experiencing recurrence after RC. Furthermore, a simple model based on those two prognostic factors demonstrated that CSS was significantly better for high-risk patients treated with SC than for similar patients treated with BSC, regardless of a history of AC. Those results underscore the potential value of 1st-line SC in patients experiencing recurrence after RC, even in the era of ICIs.

## Data Availability

The datasets used or analysed during the current study are available from the corresponding author upon reasonable request.

## Abbreviations

AC: Adjuvant chemotherapy
BSC: Best supportive care
CSS: Cancer–specific survival
CI: Confidence interval
CC: Cytotoxic chemotherapy
GC: Gemcitabine–cisplatin
HR: Hazard ratio
ICI: Immune checkpoint inhibitor
IQR: Interquartile range
mUC: Metastatic urothelial cancer
MVAC: Methotrexate–vinblastine–doxorubicin–cisplatin
RC: Radical cystectomy
SC: Salvage cytotoxic chemotherapy
